# The Implementation Research and Evaluation section: scope and future

**DOI:** 10.1093/heapol/czag103

**Published:** 2026-10-19

**Authors:** Jiangyun Chen, James R Hargreaves, Euphemia L Sibanda

**Affiliations:** Acacia Lab for Implementation Science and Center for World Health Organization Studies, School of Health Management, Southern Medical University, Guangzhou, Guangdong 510515, China; Department of Public Health, Environment and Society, Faculty of Public Health and Policy, London School of Hygiene and Tropical Medicine, Keppel Street, London WC1E 7HT, United Kingdom; Sexual and Reproductive Health & Maternal, Newborn, and Child Health (SRH & MNCH), Centre for Sexual Health and HIV AIDS Research (CeSHHAR) Zimbabwe, 10 Coronation Avenue, Greendale, Harare, Zimbabwe; Department of International Public Health, Liverpool School of Tropical Medicine, Pembroke Place, Liverpool L3 5QA, United Kingdom

As highlighted in the 40th anniversary editorial (Mills et al. 2026), evaluation has been central to *Health Policy and Planning* (HPP) since its founding in 1986, reflecting the journal’s long-standing commitment to informing the translation of evidence into policy and practice in low- and middle-income countries (LMICs). The formal establishment of the Implementation Research and Evaluation (IRE) section 14 years ago added to the journal’s scope in this regard—and while all four subject sections of the journal share this core mission (leading to blurred boundaries), each operates through a distinct analytical lens. Health Policy Processes analyses health policy processes at local, national, regional, or global levels; Health Systems Research examines health systems through a perspective of complex systems; Health Economics applies economic techniques and concepts to address health policy and system challenges; and the IRE section is dedicated to using rigorous methods to evaluate the implementation and effectiveness of interventions, policies, and programmes in real-life LMIC settings. It seeks to provide the evidence needed to ensure interventions and services are effectively designed, widely accessible, and successfully adopted by intended populations. While the section values rigorous implementation science frameworks and methods, it prioritizes real-world process evaluations, feasibility studies, and operational research designed to solve delivery bottlenecks in practice. Below, we review the past work published in this section, and set out new directions for its future.

## Key thematic contributions of the past decade

Over the past decade, our section has published more than 270 articles, with particular depth in four major domains—health workforce, maternal and child health, health financing, and digital health—addressing both persistent operational challenges and emerging priorities in LMICs. Below, we examine these thematic contributions through the lens of our section’s core focus, highlighting how scholarship in each theme informs implementation and service quality.

Primary health workforce management has remained central to our published body of work. Rather than treating personnel as passive inputs, authors show how supportive supervision, performance management, and tailored incentives directly enhance motivation and implementation fidelity across LMICs (Vasan et al. 2017, Kok et al. 2018). Studies range from syntheses of health worker performance management and national community health worker frameworks (Murphy et al. 2021) to the pragmatic scale-up of task-shifting strategies in surgical care (Bognini et al. 2023) and primary mental health services (Spagnolo et al. 2020). Additionally, published work evaluates targeted efforts to reduce provider-level stigma in service delivery (Busza et al. 2019) and practical approaches to foster respectful care behaviours among clinical personnel (Singh et al. 2021).

Real-world implementation and sustainability of maternal and child healthcare interventions have been widely reported, with scholarship examining how intervention packages behave under operational bottlenecks and systemic shocks. Syntheses of literature have mapped factors influencing maternal death surveillance (Kinney et al. 2021), complemented by field evaluations of emergency obstetric care training (Ameh et al. 2019) and qualitative investigations of mortality reviews (Melberg et al. 2019). Recent work has extended to nationwide strategy rollouts (Joe et al. 2022) and the resilience of user-fee exemption policies during crises (Aye et al. 2024)—examining operational bottlenecks at the facility level rather than macrofinancial policy design.

The section has also covered health financing architectures, strategic purchasing reforms, and provider quality accountability. While papers in the Health Economics section often explore financing mechanisms, research published in the IRE section focuses on the context-specific barriers and enablers to implementing reforms at the point of care. Landmark studies illustrate this evaluative focus by deploying standardized patients to assess clinical care quality in rural primary care (Sylvia et al. 2015) and evaluating the impact of public health insurance expansions on real-world financial protection (Grogger et al. 2015). Specifically, they move beyond knowledge or nominal coverage to examine the actual practice gaps and system constraints that determine how reforms work on the ground. Recent scholarship deepens these evaluative inquiries to examine systemic primary healthcare overhauls (Cai et al. 2023) and citizen-led social accountability frameworks evaluated specifically as implementation strategies that alter provider–community interactions to improve service delivery (Schaaf et al. 2017).

Cross-cutting innovations in digital health implementation and remote evaluation methodologies have also gained prominence. Authors have evaluated mobile health platforms for tracking routine care utilization (Ruton et al. 2018) alongside the pragmatic adaptation of everyday communication tools, such as WhatsApp Messenger as a low-cost platform for health research and field data collection (Manji et al. 2021). The IRE section has, moreover, carved out a distinct niche for pragmatic research design. Crucially, during the coronavirus disease 2019 (COVID-19) pandemic, published studies provided practical guidance on maintaining evaluative rigour under field disruptions. This included establishing ethical standards for remote fieldwork (Hensen et al. 2021) and evaluating survey tool validity through cognitive interviewing (Scott et al. 2021).

In 2020, we published a supplement (Sheikh et al. 2020) that showcased innovations in implementation research in LMICs. Although the supplement included content of relevance across all HPP sections, key highlights for the IRE section included the diversity of questions considered, alongside advances in study designs and research methods. This supplement underscored the importance of theories and frameworks and their role in generating knowledge on how implementation processes can be improved. It also shed light on the influence of contextual factors, including how local operational dynamics could impede intervention delivery, highlighting the strong role of implementation research in understanding how context shapes feasibility. Of note, the supplement emphasized the growing importance of aligning implementation research with the needs of health system decision-makers and implementers. This has spearheaded advances in collaboration through embedded research, co-creation, and power sharing with local partners to adapt programme delivery. The IRE section has continued to document how these innovations have evolved.

## From isolated pilots to embedded implementation research

A 14-year review of our section shows a clear evolution in how health delivery is evaluated across LMIC settings. Scholarship has moved from measuring the efficacy of standalone pilots of interventions towards understanding the adaptive mechanics of real-world implementation. Guided by structured frameworks, such as the Consolidated Framework for Implementation Research, Expert Recommendations for Implementing Change, and hybrid designs, contributors no longer simply ask ‘whether’ an intervention works under ideal conditions. In line with broader health policy and systems research, researchers now examine ‘how, for whom, and through what strategies’ delivery succeeds or falters within complex health systems (Adewuya et al. 2026).

A synthesis of employed methodologies in our portfolio reflects this pragmatic shift. Process evaluations account for the largest share of our published work (41%), alongside nonrandomized impact evaluations (20%), literature reviews (13%), and methodological advances (12%). Quantitative designs predominate (61%), while mixed-methods design (24%) and qualitative approaches (15%) provide essential context to unpack implementation fidelity, feasibility, and local adaptation. This profile illustrates a fruitful bridge between the World Health Organization/Special Programme for Research and Training in Tropical Diseases (WHO/TDR) tradition of ‘Implementation Research’, which focuses on frontline problem-solving, and the formal frameworks of ‘Implementation Science’ (IS). While IS tools bring valuable theoretical clarity, we emphasize balancing theory with practical field realities to avoid overly complex frameworks that prove rigid in resource-constrained settings.

In parallel, evaluation designs have transitioned from one-off surveys towards dynamic, embedded learning platforms. Harnessing routine health information systems, resilient digital tools, and process indicators, our portfolio increasingly captures delivery fidelity, equitable intervention reach, and service continuity amidst acute global shocks and climate threats (Hensen et al. 2021, Urassa et al. 2026).

## Navigating contemporary contextual challenges

Health policy, systems, and implementation research across LMICs is at a moment of change. Some of this is positive, including growing momentum to shorten the long lag between innovation and its translation into policy and practice. Also, we are in an era of digital innovations and artificial intelligence (AI) for public health, although we must be cautious about the potential dangers (Reid et al. 2025). There is growing appreciation among global and national policy makers of user-centred and community-led models of service delivery, highlighting the need for solid evaluations to help scale up effective, context-relevant care. However, the field faces challenges in how evidence is generated, funded, structured, and applied in practice.

Efforts to decolonize global health have urged reflection on the role of northern institutions in relation to health and health research in LMICs, including medical publishing. Abimbola’s (2019) recent work critiques trials of complex interventions, raising concerns that this approach illustrates the foreign gaze of global health research. This connects with ongoing debates in HPP on methodological pluralism, positivism, complexity, and realism in evaluating programmes and policies (Voller et al. 2022, Van Belle and Marchal 2025). The IRE section welcomes evaluations and trials where appropriate, if directly aligned with HPP policy to reduce the ‘foreign gaze’ by prioritizing local ownership, researcher reflexivity, and equitable authorship.

Upheaval in financing for global health and international aid, reducing official development assistance, and growing calls for LMICs to transition towards domestic self-reliance also have knock-on effects for implementation research and evaluation (Ten Brink et al. 2025). Implementation research faces a critical funding shortage, marked by recent defunding from the US National Institutes of Health (Beidas et al. 2025) and shrinking funds from other global health funders. This creates a disconnect with the growing demand for implementation evidence to inform policy and practice, stiffening competition for the fewer remaining resources. Commissioned evaluation remains a critical part of the landscape and often uses the language and methods of implementation research. Yet such evaluations serve the commissioner and are rarely published in peer-reviewed journals, limiting the published evidence base needed to drive policy change.

Health systems face various crises, ranging from disease outbreaks and conflict to climate change and antimicrobial resistance. To address these everyday and global crises, health system resilience must move beyond theory into practice. It must transcend rhetoric to become a tangible operational capacity, rather than being activated only during emergencies (Varallyay et al. 2020, Mkrtchyan et al. 2025). Across health policy and systems research, implementation research needs to show how everyday mechanisms help health systems absorb shocks and adapt over time, including through decentralized governance, intersectoral collaboration, and social accountability.

## Strategic priorities for the Implementation Research and Evaluation section

IRE studies pose specific challenges that the authors of submissions to our journal rise to in their work. As section editors, we sometimes characterize these as the ‘FURRIE’ challenges (feasible, useful, rigorous, relevant, informative, and equitable) (Hargreaves et al. 2024). Delivering evaluations in real-world settings is never easy, and there are important barriers that evaluators must overcome in making studies **F**easible to undertake and generate the evidence that is needed. Identifying and overcoming these barriers is critical. Evaluations must be judged on their **U**sefulness, and we apply this lens in assessing submissions to the IRE section of the journal. Studies we publish in this section will consider and achieve the right balance between delivering **R**igorous evaluation methodology (quantitative, qualitative and mixed methods) and paying keen attention to the real-world **R**elevance of the evidence they generate. This section seeks papers (and appendices) that are detailed and **I**nformative, allowing our readers to judge what was done and to replicate or adapt the reported work. Finally, studies reported in the IRE section must pay close attention to power dynamics among stakeholders in any evaluative process, including, but not limited to, **E**quitable practices among evaluation teams and author groups.

To maintain these standards, we seek to address key methodological imbalances in incoming submissions. We still receive many papers reporting on small-scale studies that largely use descriptive methods. Over the years, we have also seen growing numbers of papers reporting on context/setting-specific evaluations that fail to extract translatable lessons for audiences outside the country of origin. We encourage authors to move beyond listing broad barriers towards focused inquiries guided by clear hypotheses and analytical frameworks. Submissions should distinguish the core health interventions from the strategies used to implement them. To make these strategies replicable, we encourage the use of standardized reporting tools, such as TIDieR (Hoffmann et al. 2014), and preregistration of study protocols in public registries to increase transparency. Although HPP does not publish standalone protocols, primary research backed by preregistration is strongly welcomed by the IRE section.

Looking ahead, we align our strategic vision with four core priorities that address pressing global realities. These priorities adapt the major contextual challenges identified in the Health Systems section editorial (Spicer et al. 2026) into a specific focus for the IRE section. While the Health Systems section examines these forces through a governance lens, our section focuses on how health interventions are delivered, adapted, and evaluated in real-world settings. First, the expansion of digital tools and AI requires evaluations measuring their impact and equity in health delivery. Second, climate shifts create new challenges for health service delivery and community health, requiring research on how services adapt to maintain care. Third, process evaluations need to capture how structural forces, such as resource allocation and power dynamics, influence programme implementation and effectiveness. Fourth, political instability and financial shifts require evaluations assessing how health services adapt to funding changes, with close attention to whether quality or equity degrades, while ensuring power sharing with local partners.

To achieve these goals, while process evaluations remain our foundational strength, we actively seek to publish more high-quality pragmatic trials. This section also welcomes rigorous impact and outcome evaluations explicitly connecting implementation strategies to health outcomes, which currently account for around 25% of our published work. Furthermore, while Commentaries (8%) and Innovation and Practice Reports (1%) currently represent a small share of our publications, we encourage well-substantiated submissions on topical policy issues that offer lessons beyond a single country. Indeed, we have seen a clear increase in mixed-methods studies over the last 5 years (from 8% in 2022 to 43% as of mid-2026). In addition to these percentages, we have seen a growing trend of submissions of primary research studies that use theory-based methods for intervention evaluation, e.g. realist evaluation models that seek to explore how, why, and for whom an intervention works. These provide relevant information that policy makers and programmes in LMICs can use to tailor evidence-based interventions to their contexts. In these studies, context should not be treated merely as a descriptive background, but as a central factor that actively shapes implementation. We also plan to commission more papers focused on optimizing service delivery and scaling up evidence-based interventions.

Crucially, we challenge researchers to look beyond standard measures of success, such as coverage and health outcomes, to include user experience, cultural and contextual appropriateness, and equity. Studies reporting implementation failures, unexpected bottlenecks, or negative findings are equally valuable, as they provide critical lessons for policy and practice. Finally, we welcome research on long-term sustainability and practical approaches that bridge the gap between evidence and practice, helping move beyond short-term pilot projects of implementation strategies.

The HPP IRE section has achieved great success in the last 14 years, publishing high-quality papers with important implications for policy and practice. It is important that the section is responsive to the contextual challenges facing countries around the world by promoting submission of manuscripts in these topical areas. Importantly, to foster improvement in the robustness and impact of the implementation research studies HPP publishes, we encourage health programme and implementation researchers to address the six FURRIE challenges during intervention development, implementation, and reporting.

## Data Availability

Not applicable for this editorial.
